# Scraps

**Published:** 1900-09

**Authors:** 


### SCRAPS

PICKED UP BY L. M. G.

**Clinical Records** (32).—First Oculist: "I had a most interesting case yesterday." Second Oculist: "What was it?" First Oculist: "I saw a young lady who instead of a common pupil has a college student in her eye."

**Medical Meetings.**—Dr. J. N. Hall, in the *Journal of the American Medical Association*, says: "Did you ever study why it was that some physicians fail to attend the meetings of the medical societies? Perhaps it begins in carelessness or laziness, but sooner or later such men are likely to get so far behind their brethren that they do not attend because they are painfully conscious of the fact and fear to appear at a disadvantage. The physician who does not associate with his fellows runs great risk of falling into one of two errors. Either he becomes an egotist, because he fails to see the good work that others are doing, or, if of a timid nature, and faithful in his work, from knowing his own weaknesses so well, he feels that he is immeasurably behind his brothers in the profession. One cannot see where he stands in the race unless he sees both those in front of him and those in the rear. Let us, then, support warmly our medical societies. Let us inculcate into the minds of the younger men all that is best of the sacred inheritance which has come down to us from the earliest times, that they may know the debt they owe to the profession, and be led to attempt to add something to our store of knowledge, as an acknowledgement of the immensity of the debt. Let us instill into them ideals so high that they shall make of our occupation, not a mere trade, but what it should be, a liberal profession, and, I thank God, the noblest profession it is given to the sons of men to follow."

**"The Apothecary Poet."**—In *The Asclepiad* for 1884 Dr. B. W. Richardson had a biographical record of Keats, embellished with a very excellent portrait. Dr. William Osler has also an interesting article on Keats in the *Johns Hopkins Hospital Bulletin* for January, 1896. Other authorities for the life and work of Keats are too well known to need specific mention.

Keats, after being a student at the combined schools of Guy's and St. Thomas's, passed the Apothecaries' Hall in 1816, but gave up medicine in the following year. On September 23rd, 1819, in writing to his friend, C. A. Brown (the author of *Shakespeare's Autobiographical Poems*), he says: "In no period of my life have I acted with any self-will but in throwing up the apothecary profession."

That Keats and Brown had each a pretty wit may be seen from what occurred concerning a house which Brown had let to a Nathan Benjamin. The water of the house came through a tank which was lined with lime. Wishing to have a joke with Brown, Keats wrote the following note in Benjamin's name:—

"Sir,—By drinking your damn'd tank water I have got the gravel. What reparation can you make to me and my family? "Nathan Benjamin."

Brown accordingly surprised his tenant with the following answer:—

"Sir,—I cannot offer you any remuneration until your gravel shall have formed itself into a stone, when I will cut you with pleasure. "C. Brown."

In the summer of 1818 Keats began to get throat trouble, no doubt the beginning of the tuberculosis from which he died. On February 3rd, 1820, he had an attack of hæmoptysis. From this date we can trace in his letters the melancholy progress of the disease. On June 22nd he had a return of the spitting of blood, which lasted several days. The serious nature of the disease was by this time evident to both the patient and his physicians. He acknowledges that it will be a long, tedious affair, and that a winter in Italy may be necessary. "'Tis not yet consumption," he writes Fanny Keats, "but it would be were I to remain in this climate all the winter." This, too, was a time of terrible mental distress, as he became madly jealous of Brown. The letters of this period to Fanny Brawne tell of the "damned moments" of one who "dotes yet doubts, suspects yet fondly loves." Preparations were made for his journey to Italy, which he speaks of "as marching up to a battery." He sailed for Naples, which was reached after a tedious voyage about the end of October, 1820. Severn, the artist, accompanied him, and has given (*Atlantic Monthly*, April, 1863) a touching account of the last months of his friend's life. In Rome, Keats was under the care of Dr. (afterwards Sir) James Clark, who, with Severn, watched him with assiduous care throughout the winter months. On February 14th, 1821, he requested Severn to have inscribed on his grave-stone the words: "Here lies one whose name was writ in water"; and on the 23rd, not having reached his twenty-sixth birthday, he passed away quietly in Severn's arms.

**Medical Philology** (XXXV.).—The *Promptorium* gives "Dragaunce, herbe. *Dragancia*, *basilica*, *dracentra*," and the *Catholicon* "Dragence or nedd*er* grysse; *dragancia*, *basilisca*, *herba serpentaria vel serpentina*." In his note Mr. Way says: "Numerous virtues are ascribed by Macer and other writers to the herb dragaunce or nedder's tongue, called also dragon wort, addyrwort, or serpentine, arum or aron. See Roy. MS. 18 A. VI. f. 73. Macer says that 'water of dragaunce ys gode to wasshe venome soris,' and it appears to have been yearly distilled in the household of the Earl of Northumberland, 1511. See Antiqu. Rep. iv., 284." Mr. Herrtage's note gives "'*Dracontium*. Dragon wort or dragens.' Cooper. Cogan, Haven of Health, 1612, p. 72, recommends the use of *Dragons* as a specific for the plague. Harrison, Descript. of England, ii. 34, says that the sting of an adder brings death, 'except the iuice of *dragons* (in Latine called *Dracunculus minor*) be speedilie ministred and dronke in stronge ale.'"

Dragonwort, the obsolete name of the *Dracunculus vulgaris*, was reputed not only to be useful, as Harrison said, for the cure of the sting of an adder, but was credited with prophylactic power, for Lyte, *Dodoens*, III. vi. 322 (quoted in the *New English Dictionary*), says: "It is thought . . . that those which carrie about them the leaues or rootes of great Dragonwurtes, cannot be hurt nor stong of Vipers and Serpentes."

Our forefathers must have been satisfied with very slender evidence of the curative qualities of remedies which they vaunted highly and with much assurance. It would seem that they considered that, having received the name "nedder's tongue" or "addyrwort" on account of the shape of portions of the plant, it must have a beneficial effect in troubles arising from the animal which it was supposed to represent. Adder's tongue, which as a name should be restricted to the fern *ophioglossum*, was "applied loosely to various other plants, superficially more or less resembling it."

The form "nedder" is interesting as a fifteenth century instance of the original form which "lost its initial *n* through the erroneous division of *a naddre* as *an addre*." Apron, orange, and umpire are some other instances of English words having lost their initial *n*. The *Promptorium* has "Barmclothe,[1] or naprun. *Limas*," "Eddyr, or neddyr, wyrme. *Serpens*" and "Nowmpere, or owmpere. *Arbiter*, *sequester*." As it was thought that it meant "the *golden* fruit," orange lost its *n* very early. It appears only in that form in the *Promptorium*. There is a record that the fruit so named came to England in 1290. On the other hand, the word newt has permanently gained its initial from the final letter of *an*, and other words borrowed it for a time. The *Catholicon* has "a Nere; Auris, auricula," and Mr. Herrtage in his note quotes "*Hec auris*, *A*$^{e}$ nere" from Wright, who in his *Vocabularies* says that the practice of prefixing the final *n* of an article to the noun, when the latter commences with a vowel, is of constant recurrence in vocabularies of the fifteenth century. The Rev. Walter W. Skeat, in his *Etymological Dictionary*, names some of these, and refers to Halliwell's *Dictionary of Archaic and Provincial Words* for examples of them. In the Rev. A. Smythe Palmer's *Folk-Etymology*, the twenty-four pages on "Words Corrupted by Coalescence of the Article with the Substantive" afford much of interest and erudition on this subject.

Dragon is used in a great variety of combinations, probably always with the idea of special force or strength or efficacy. That which is known commercially as dragon's blood has some medical interest, as it is the styptic I have often seen the Jewish minister use for his circumcisions. It is a resinous substance generally obtained from the fruit of *Calamus Draco*.

[1] Barm, meaning bosom or lap, is often met with from the tenth to the fifteenth century. Chaucer has it in several places.

